# Integrative analysis of transcription factors and microRNAs in ovarian cancer cell spheroids

**DOI:** 10.1186/s13048-020-00618-7

**Published:** 2020-02-11

**Authors:** Hyun Park, Sohyun Hwang, Ju-Yeon Jeong, Sang Geun Jung, Min Chul Choi, Won Duk Joo, Seung Hun Song, Chan Lee, Hee Jung An

**Affiliations:** 1 Division of Gynecologic Oncology, Department of Obstetrics and Gynecology, College of Medicine, CHA University, Seongnam, Gyeonggi-do South Korea; 2 Department of Pathology, College of Medicine, CHA University, Seongnam, 351 Yatap-dong, Bundang-gu, Seongnam-si, Gyeonggi-do 13496 South Korea; 3 Institute of Clinical Research, College of Medicine, CHA University, Seongnam, Gyeonggi-do South Korea

**Keywords:** Ovarian epithelial carcinoma, Spheroid-forming cells, Cancer stem cells, Transcription factors, microRNAs

## Abstract

**Background:**

Cancer stem cells (CSCs) can self-renew, proliferate into differentiated cells, or enter a quiescent state and are regarded to cause chemoresistance and recurrence. An integrative analysis of transcription factors (TF) and miRNAs was performed in ovarian CSC-enriched spheroid-forming cells (SFCs) to identify factors relevant to ovarian CSCs.

**Methods:**

Fresh tumor cells from three ovarian cancer patients were cultured in standard and in selective medium. The mRNAs and miRNAs that exhibited significant differential expression between SFCs and adherent cells were identified using mRNA and miRNAs microarrays. Target genes of miRNAs were further selected if predicted with TargetScan by half of the miRNAs or more. Gene enrichment analysis was performed on over- or under-expressed mRNAs and target genes of miRNAs using DAVID tools. Complex regulatory networks were combined from TF-genes and miRNA-genes interactions using the MAGIA webtool.

**Results:**

A total of 1245 mRNA and 55 miRNAs were differentially expressed (*p*-value< 0.05, paired t-test). Elevation of transcription-related processes and suppression of focal adhesion pathway were noted in SFCs, according to the enrichment analyses. Transcriptional hyperactivity is a known characteristic of the stem cell transcriptome. The integrative network suggested that cell cycle was arrested in SFCs where over-expressed EGR1 and under-expressed MYC and miR-130a-3p had multiple connections with target genes.

**Conclusions:**

MYC, EGR1, and miR-130a-3p were hubs in our integrative analysis of ovarian CSC-enriched SFCs, suggesting that ovarian cancer SFCs display a stem cell identity with the quiescent phenotype where adhesion- and cell cycle-related genes were suppressed.

## Background

Ovarian cancer is a devastating gynecologic malignancy. Most patients are diagnosed at an advanced stage, and are vulnerable to recurrence of the disease. About 70% of cases have intraperitoneal dissemination at initial diagnosis [1]. These cases usually regress completely following primary cytoreductive surgery and adjuvant chemotherapy targeting residual disease. However, most patients experience recurrence, which suggests the presence of chemoresistant microlesions.

Cancer cell aggregates or spheroids are an important step in metastasis and cell survival in chemotherapy [2]. After ovarian cancer cells are shed from the primary tumor, they grow as spheres floating in ascites and disseminate through the peritoneal cavity [3]. Spheroids are proposed to mainly consist of cancer stem cells (CSCs) which have potential to evade therapy [4]. Additionally spheroids in this non-adherent condition enter a quiescent or dormant state, a temporary arrest of proliferation, and become refractory to chemotherapy [5].

Cellular quiescence is genetically controlled by a combination of environmental cues from stem cell niche and cell intrinsic factors especially associated with cell cycle and transcriptional regulation [6, 7]. MiRNAs are well-known regulators in numerous biologic processes including proliferation and metastasis. Some miRNAs are reported to govern the phenotypes of tumors such as outgrowth or prolonged dormancy [8].

In this study we examined and integrated the mRNA expression of transcription factors and miRNA expressions of spheroids derived from primary ovarian cancers to identify factors regulating ovarian cancer stem cells. The key regulators and their functions were reviewed in terms of stem cell features, which may present relevant targets for novel therapeutics to reduce treatment resistance and recurrence of ovarian cancer.

## Materials and methods

### Patients and tissue samples

Tissues were sampled from specimens obtained from staging operation including oophorectomy for high grade serous adenocarcinoma of ovary. A total of five patients were initially enrolled, however three corresponding sets from 3 patients were studied for matched analysis of mRNA and miRNA expression because one patient was proved to be low grade serous carcinoma, and one sample did not pass the RNA QC for microarray. The clinicopathological characteristics of the cases were listed on Additional file 1: Table S1. Informed consent was obtained from the patients before surgery. This study was approved by the Ethical Committee of CHA Bundang Medical Center (CHAMC 2009–019).

### Primary cell culture and spheroid-forming cell (SFC) isolation

Tumors were mechanically dissected into small pieces and enzymatically digested at 37 °C for 1 h into single-cell suspensions using collagenase A (50 U/mL, Roche, Basel, Switzerland) contained in Ca/Mg-free phosphate-buffered saline. Cells were incubated with Ber-EP4-coated magnetic Dynabeads (Life Technologies, Grand Island, NY) for 30 min to select epithelial cells, which were then cultured in RPMI medium (Gibco/Life Technologies, Grand Island, NY) containing 10% fetal bovine serum, 1% penicillin-streptomycin, and 20 ng/mL epidermal growth factor (Life Technologies).

For spheroid formation, single cells were plated on ultra-attachment six-well culture plates (Corning, Acton, MA) at a density of 1 × 10^3 cells/cm^2^ in serum-free Dulbecco’s modified Eagle’s medium/F12 medium (Life Technologies) supplemented with 20 ng/mL epidermal growth factor (Life Technologies), 10 ng/mL basic fibroblast growth factor (Sigma-Aldrich), and 5 μg/mL insulin (Sigma-Aldrich). Spheroid formation of 50–100 cells was assessed at 7 days after seeding.

### RNA extraction

Cultured SFCs were passed through a tube installed with nylon mesh of 35 μm pore-size. Only the globular SFCs on the mesh were collected and pelleted to remove the media. RNA was isolated from SFCs and corresponding primary cancer cells at the same passage (passage2) using TRIzol reagent (Life Technologies), according to the manufacturer’s instructions. Total RNA quantification was performed using a Nanodrop spectrophotometer (NanoDrop Technologies, Inc., Wilmington, DE, USA). The integrity of the isolated RNA and miRNA was examined by OD 260/280, OC260/230, 28S/18S ratio and RNA integration number (RIN) using Agilent 2100 Bioanalyzer. The RNA quality was listed up in Additional file 1: Table S2. All 3 matched samples, which passed the RNA QC, were referred to microarray analysis.

### cDNA microarray analysis

Synthesis of target cRNA probes and hybridization were performed using Agilent’s Low RNA Input Linear Amplification kit (Agilent Technology, USA). Briefly 25 ng total RNA was reversely transcribed to dsDNA with cDNA master mix. Then, dsDNA were labelled with Cyanine 3-pCp (Cy3) or Cyanine 3-pCp (Cy5) and transcribed to cRNA according to manufacturer’s protocol. Amplified and labeled cRNA was purified on the cRNA Cleanup Module (Agilent Technology). Each 850 ng of Cy3-labeled and Cy5-labeled cRNA target were mixed and fragmented for hybridization. The fragmented cRNA was pipetted directly onto assembled Human Oligo Microarray (60 K) (Agilent Technology).

Hybridized images were scanned using a DNA microarray scanner and quantified with Feature Extraction Software (Agilent Technology). Data normalization and selection of significantly changed genes was performed using GeneSpring GX 7.3 (Agilent Technology). Intensity-dependent normalization (LOWESS) was performed, where the ratio was reduced to the residual of the Lowess fit of the intensity vs. ratio curve. The averages of normalized ratios were calculated by dividing the average normalized signal channel intensity by the average normalized control channel intensity.

### microRNA microarray

MicroRNA microarray analysis was performed as instructed by the manufacturer. One hundred nanograms of RNA from each sample was labeled with Cy3 using Agilent’s miRNA Complete Labeling and Hyb Kit. The Cy3-labeled RNAs were hybridized to the miRNA microarray (Agilent Human miRNA 8*60 K, Rel 18.0). The miRNA microarray was then scanned using the Agilent G2600D microarray scanner. Raw data for the same gene in primary ovarian cancer cells and SFCs were summarized in the Agilent Feature Extraction software package (v11.0.1.1), which generated the gene view file and provided expression data for each gene probed on the array. Array data were filtered using gIsGeneDetected = 1 for all samples (1: detected). Logarithmically transformed miRNA gtotalGeneSignal values were normalized with the quantile method [9]. R statistical language software package (v. 2.15.0) performed the normalize.quantiles function of the preprocessCore package (https://www.rdocumentation.org/packages/preprocessCore/versions/1.34.0/topics/normalize.quantiles). The comparative analysis of results from primary cancer cells and SFCs was based on fold changes.

### Gene enrichment analysis

mRNAs and miRNAs that had been differently expressed between spheroid and parental cells with significance (*p*-value< 0.05, paired t-test) based on microarray data were included in the analysis. Target genes of miRNAs were identified with TargetScan (http://www.targetscan.org) and further selected if they were predicted by at least half of the miRNAs. The gene list was submitted to the Database for Annotation, Visualization, and Integrated Discovery (DAVID), version 6.7 (http://david.abcc.ncifcrf.gov/) and annotated by Gene Ontology enrichment analysis and Kyoto Encyclopedia of Genes and Genomes (KEGG) pathway enrichment analysis (https://www.genome.jp/kegg/). The pathway enrichment was regarded to be significant when the *p*-value of modified Fisher’s exact test was less than 0.05. .

### Integrative network analysis

**Fig. 1:**
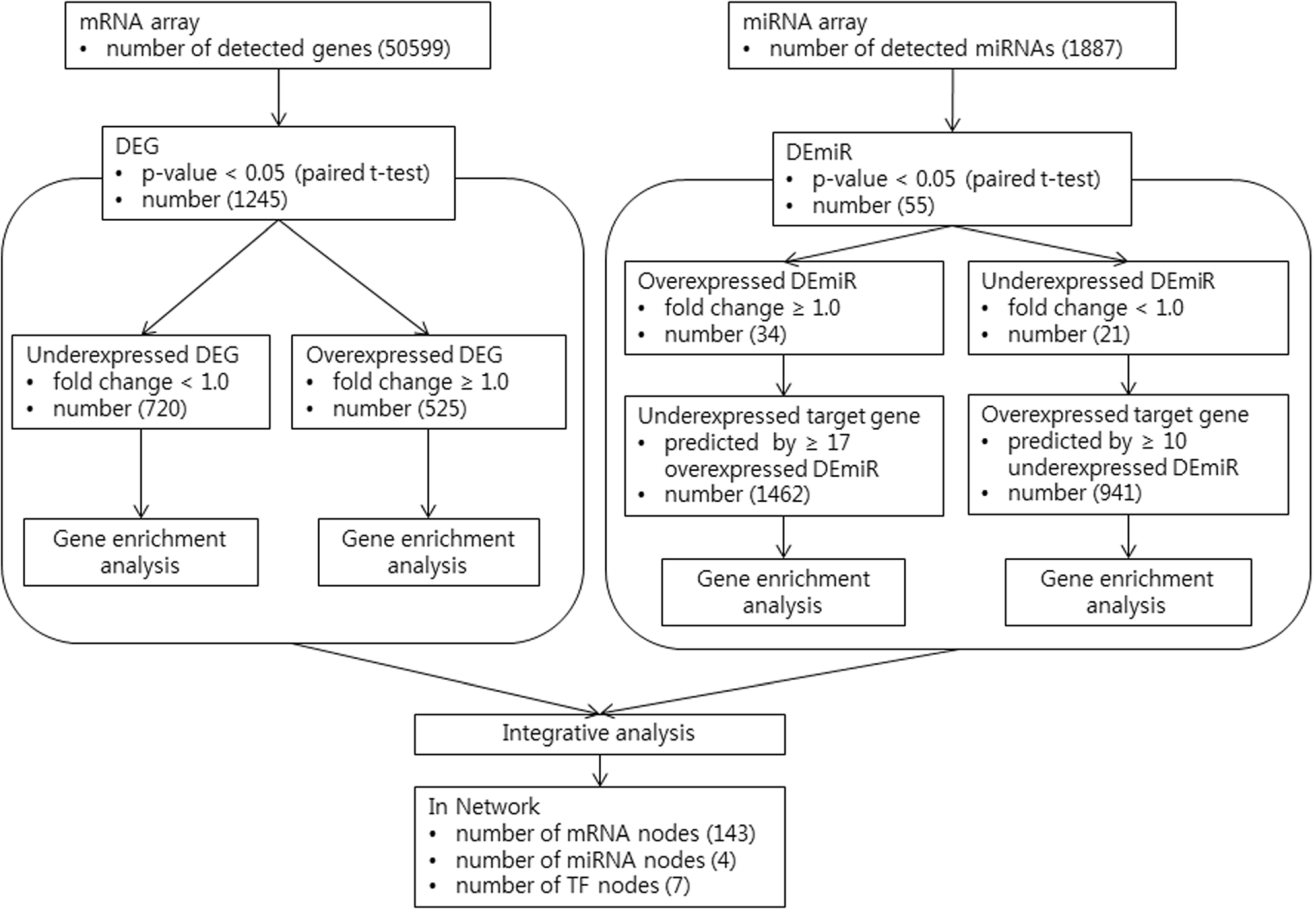
Flow of data for integrative analysis with mRNAs and miRNAs. DEG and DEmiR were selected from microarray data (paired t-test, *p*-value < 0.05), and targets of DEmiR were predicted with TargetScan. At first four groups of over- and under-expressed genes/target genes were analyzed independently to identify the activated or suppressed functions of SFCs. Thereafter, integrative analysis of DEG and DEmiR was performed using the MAGIA^2^ webtool. DEG: differentially expressed gene, DEmiR: differentially expressed miRNA, SFC: spheroid-forming cell

The data flow for integrative analysis is summarized in Fig. 1. The probe lists of genes and miRNA with statistical significance (p-value< 0.05, paired t-test) were processed and uploaded to each matrix. All differentially expressed probes were included in the selection criteria in order to identify the effects of under-expressed regulators. The integrative analysis was conducted with MAGIA2 (http://gencomp.bio.unipd.it/magia2), which is a web application incorporating in silico target prediction, miRNA, and gene expression data [10]. The whole results were downloaded as tab delimited files which describe TF-target and miRNA-target data. Cytoscape was used to import and merge the files in order to re-visualize the network [11]. The activity of biologic processes of interest was evaluated with chi-square test which analyzes the relationship between the expression level of genes (low vs. high group) and a particular process to which the genes belong (one process vs. others group).

## Results

### Characteristics of isolated primary carcinoma cells and spheroid-forming cells

After tumor cells isolation, the immune cells comprised about 10–15% of the whole isolated cells. After selection with Ber-EP4-coated magnetic dynabeads, the immune cells were less than 1% (FACS data, Additional file 1: Figure S1). Therefore, the cultured cells were said to be purely carcinoma cells.

The nonadherent spherical clusters of 50–100 cells were observed 1 week after plating on spheroid-forming assay. The efficiency of spheroid formation from the inoculated cells was 2.4% ± 0.2% in the first generation. The spheroid-forming capacity in the second generation was similar to the first (2.3% ± 0.3%) [12].

### Differential expression of mRNA and miRNA

In SFCs compared with parental cancer cells, 1245 genes were differentially expressed (*p* < 0.05), with 720 (57.8%) under-expressed and 525 (42.2%) over-expressed. For miRNAs, 55 were selected which were differentially expressed (*p* < 0.05) where 21 (38.1%) were under-expressed and 34 (61.9%) were over-expressed in SFCs. A total of 921 and 1420 target genes were listed that had been predicted multiple times by more than 10 under-expressed miRNAs and 17 or more over-expressed miRNAs, respectively. The data of mRNA and miRNA microarray can be accessed through Gene Expression Omnibus (GEO) Series accession number GSE136924 and GSE136925 respectively (www.ncbi.nlm.nih.gov/geo).

The functions of those genes were analyzed using DAVID for gene enrichment analyses (Tables 1, 2, 3, 4, 5, 6, 7 and 8). Transcription, DNA-templated was the most significantly altered biologic process in both over-expressed mRNAs (false discovery rate, FDR = 5.3E-8) and target genes of under-expressed miRNAs (FDR = 3.9E-3). According to KEGG pathway analysis, focal adhesion (FDR = 2.3E-4) and ErbB pathways (FDR = 6.2E-3) were affected the most in under-expressed mRNAs and target genes of over-expressed miRNAs, respectively, which were suppressed in SFCs.

**Table 1:** Gene ontology annotated from over-expressed genes in spheroid cells compared with parental cells

Term	Count	%	*P*-Value	Bonferroni
GO:0006351~transcription, DNA-templated	96	18.3	2.99E-11	5.28E-08
GO:0006355~regulation of transcription, DNA-templated	73	13.9	2.40E-08	4.23E-05
GO:0000122~negative regulation of transcription from RNA polymerase II promoter	34	6.4	4.40E-04	0.540151
GO:0045893~positive regulation of transcription, DNA-templated	26	4.9	9.82E-04	0.82318
GO:0006366~transcription from RNA polymerase II promoter	25	4.7	0.001996	0.970526
GO:0010467~gene expression	6	1.1	0.006248	0.999984
GO:0051726~regulation of cell cycle	9	1.7	0.011435	1
GO:0036498~IRE1-mediated unfolded protein response	6	1.1	0.014689	1
GO:0051569~regulation of histone H3-K4 methylation	3	0.5	0.019287	1
GO:0033762~response to glucagon	3	0.5	0.023722	1

**Table 2:** KEGG pathways annotated from over-expressed genes in spheroid cells compared with parental cells

Term	Count	%	*P*-Value	Benjamini
hsa00190:Oxidative phosphorylation	9	1.7	0.006861	0.731536
hsa05016:Huntington’s disease	11	2.0	0.007159	0.496491
hsa05010:Alzheimer’s disease	10	1.9	0.008861	0.432572
hsa04260:Cardiac muscle contraction	6	1.1	0.020595	0.629784
hsa05012:Parkinson’s disease	8	1.5	0.030054	0.688289
hsa04932:Non-alcoholic fatty liver disease (NAFLD)	8	1.5	0.039968	0.727043
hsa04720:Long-term potentiation	5	0.9	0.049961	0.753023
hsa04141:Protein processing in endoplasmic reticulum	8	1.5	0.065682	0.8025
hsa04931:Insulin resistance	6	1.1	0.077341	0.818825
hsa01212:Fatty acid metabolism	4	0.7	0.079851	0.795971

**Table 3:** Gene ontology annotated from under-expressed genes in spheroid cells compared with parental cells

Term	Count	%	*P*-Value	Benjamini
GO:0006936~muscle contraction	17	2.3	9.22E-07	0.002228
GO:0060314~regulation of ryanodine-sensitive calcium-release channel activity	7	0.9	3.27E-05	0.038794
GO:0098609~cell-cell adhesion	23	3.2	2.24E-04	0.165232
GO:0010881~regulation of cardiac muscle contraction by regulation of the release of sequestered calcium ion	6	0.8	3.97E-04	0.213532
GO:0044319~wound healing, spreading of cells	5	0.7	5.85E-04	0.246555
GO:0006636~unsaturated fatty acid biosynthetic process	5	0.7	0.001483	0.450441
GO:0072661~protein targeting to plasma membrane	6	0.8	0.001834	0.46994
GO:1901380~negative regulation of potassium ion transmembrane transport	4	0.5	0.002087	0.468405
GO:0070527~platelet aggregation	7	0.9	0.002822	0.532242
GO:0010880~regulation of release of sequestered calcium ion into cytosol by sarcoplasmic reticulum	5	0.7	0.003059	0.523566

**Table 4:** KEGG pathways annotated from under-expressed genes in spheroid cells compared with parental cells

Term	Count	%	*P*-Value	Benjamini
hsa04510:Focal adhesion	25	3.5	9.56E-07	2.44E-04
hsa04810:Regulation of actin cytoskeleton	23	3.2	1.58E-05	0.002008
hsa04270:Vascular smooth muscle contraction	16	2.2	3.63E-05	0.003084
hsa05205:Proteoglycans in cancer	21	2.9	7.53E-05	0.004789
hsa04921:Oxytocin signaling pathway	17	2.3	1.89E-04	0.009584
hsa04520:Adherens junction	11	1.5	3.41E-04	0.014382
hsa04310:Wnt signaling pathway	14	1.9	0.002365	0.082638
hsa01100:Metabolic pathways	64	8.9	0.00614	0.178252
hsa00230:Purine metabolism	15	2.1	0.00754	0.193017
hsa04912:GnRH signaling pathway	10	1.4	0.007892	0.182938

**Table 5:** Gene ontology annotated from genes targeted by under-expressed miRNAs in spheroid cells compared with parental cells

Term	Count	%	*P*-Value	Benjamini
GO:0006351~transcription, DNA-templated	141	15.3	1.34E-06	0.00394
GO:0006355~regulation of transcription, DNA-templated	105	11.4	1.47E-04	0.19426
GO:0007611~learning or memory	9	0.9	2.75E-04	0.235702
GO:0060509~Type I pneumocyte differentiation	4	0.4	0.00107	0.544146
GO:0045944~positive regulation of transcription from RNA polymerase II promoter	70	7.6	0.001317	0.538713
GO:0001782~B cell homeostasis	6	0.6	0.001365	0.487511
GO:0000122~negative regulation of transcription from RNA polymerase II promoter	54	5.8	0.00178	0.526265
GO:0017148~negative regulation of translation	10	1.0	0.00184	0.491213
GO:0016055~Wnt signaling pathway	20	2.1	0.001982	0.476491
GO:0035278~miRNA mediated inhibition of translation	5	0.5	0.002021	0.447845

**Table 6:** KEGG pathways annotated from genes targeted by under-expressed miRNAs in spheroid cells compared with parental cells

Term	Count	%	*P*-Value	Benjamini
hsa05223:Non-small cell lung cancer	10	1.0	5.70E-04	0.129304
hsa04070:Phosphatidylinositol signaling system	13	1.4	9.47E-04	0.108708
hsa05231:Choline metabolism in cancer	13	1.4	0.001238	0.095495
hsa05214:Glioma	10	1.0	0.001713	0.098896
hsa04710:Circadian rhythm	7	0.7	0.001786	0.083219
hsa04713:Circadian entrainment	12	1.3	0.002413	0.093214
hsa05202:Transcriptional misregulation in cancer	16	1.7	0.005361	0.17023
hsa04550:Signaling pathways regulating pluripotency of stem cells	14	1.5	0.007051	0.193411
hsa04664:Fc epsilon RI signaling pathway	9	0.9	0.008364	0.202908
hsa05220:Chronic myeloid leukemia	9	0.9	0.011687	0.248486

**Table 7:** Gene ontology annotated from genes targeted by over-expressed miRNAs in spheroid cells compared with parental cells

Term	Count	%	*P*-Value	Benjamini
GO:0007156~homophilic cell adhesion via plasma membrane adhesion molecules	29	2.0	1.54E-05	0.056544
GO:0046777~protein autophosphorylation	30	2.1	2.90E-05	0.053211
GO:0086012~membrane depolarization during cardiac muscle cell action potential	7	0.4	1.87E-04	0.209386
GO:0006351~transcription, DNA-templated	186	13.0	3.32E-04	0.269164
GO:0045893~positive regulation of transcription, DNA-templated	59	4.1	0.001122	0.571204
GO:0007417~central nervous system development	20	1.4	0.00143	0.593301
GO:0061337~cardiac conduction	11	0.7	0.001496	0.553802
GO:0051899~membrane depolarization	8	0.5	0.001859	0.584091
GO:0018105~peptidyl-serine phosphorylation	20	1.4	0.002322	0.6226
GO:0006810~transport	42	2.9	0.002517	0.613515

**Table 8:** KEGG pathways annotated from genes targeted by over-expressed miRNAs in spheroid cells compared with parental cells

Term	Count	%	*P*-Value	Benjamini
hsa04012:ErbB signaling pathway	19	1.3	2.22E-05	0.005825
hsa04713:Circadian entrainment	19	1.3	7.62E-05	0.009974
hsa04144:Endocytosis	32	2.2	5.76E-04	0.049252
hsa04921:Oxytocin signaling pathway	23	1.6	6.27E-04	0.040372
hsa05223:Non-small cell lung cancer	12	0.8	0.001382	0.070185
hsa04725:Cholinergic synapse	18	1.2	0.001567	0.066448
hsa04720:Long-term potentiation	13	0.9	0.00172	0.062632
hsa04150:mTOR signaling pathway	12	0.8	0.001864	0.059484
hsa04068:FoxO signaling pathway	20	1.4	0.002159	0.061212
hsa04020:Calcium signaling pathway	24	1.6	0.002917	0.073956

### Network analysis

**Fig. 2:**
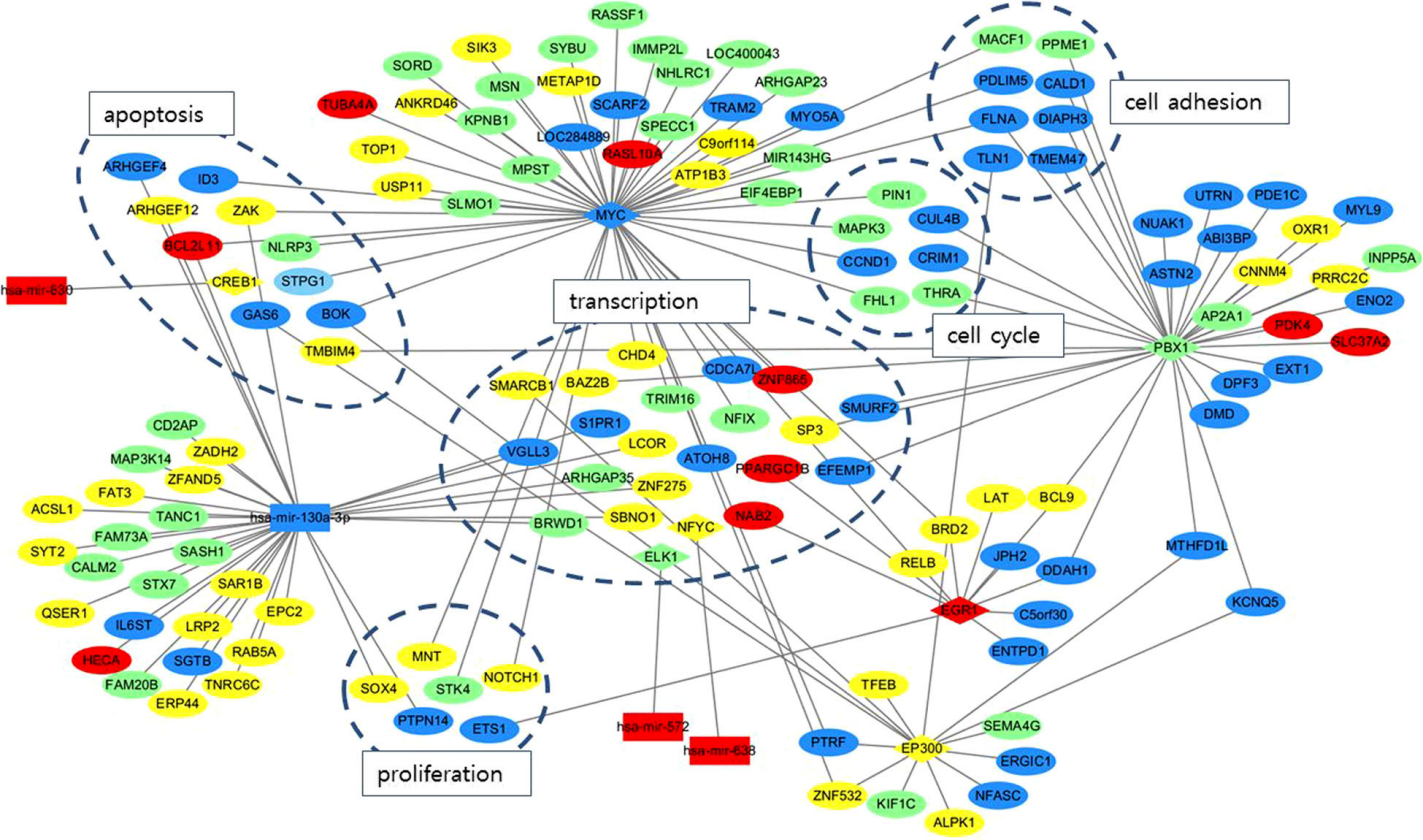
Integrative network composed of TFs, miRNAs, and target genes. Genes of known function were grouped using dotted lines. Transcription-related genes were most common, while genes of cell cycle and cell adhesion were suppressed in SFCs. EGR1, MYC, and miR-130a-3p were the main regulators to remain as a quiescent phenotype of stem cell. Rectangle: TF, ellipse: gene, diamond: miRNA, colors indicate fold change of the expression in spheroid-forming cells relative to parental cells (red: ≥2, yellow: 2 > to > 1, green: 1 > to ≥0.5, blue: > 0.5), SFC: spheroid-forming cell, TF: transcription factor

We found a total of 147 nodes and 151 interactions with FDR < 0.05 (Fig. 2). There were seven TFs and four miRNAs as regulators. While three TFs had only one interaction of TF-miRNA or miRNA-TF, four had multiple interactions with target genes of various functions, such as transcription, cell adhesion, apoptosis, proliferation, and cell cycle. There were four miRNAs where miR-130a-3p had multiple interactions with genes of transcription, apoptosis, and proliferation. Three regulators, EGR1 (fc = 2.479), MYC (fc = 0.387), and miR-130a-3p (fc = 0.381), were more differentially expressed (≥2 or ≤ 0.5-fold change (fc)) than others (2 < fc < 0.5) (Table 9).

**Table 9:** Regulators with multiple connections in network and their roles in stem cells

Regulators	Fold change	P-value	Function in tumor	Function in stem cell	Reference
EGR1	2.479	0.045	Tumor suppressor, Chemoresistance enhancer	Promote quiescence	[12], [13], [14]
MYC	0.387	0.036	Proto-oncogene	Inactivation induce quiescence or differentiation	[15, 16]
PBX1	0.759	0.032	Stem cell reprogramming factor, Chemoresistance enhancer	Maintain quiescence	[17, 18]
EP300	1.484	0.005	Tumor suppressor, Chemoresistance modulator	Modulate stemness	[19]
miR-130a-3p	0.381	0.007	Tumor suppressor, Chemoresistance modulator	Target genes maintain quiescence	[20–22]

Transcription-related genes were the most common in the network that combined upregulated and downregulated target genes. The genes involved in cell adhesion (chi-square test, *p* = 0.019) and cell cycle (*p* = 0.043) were significantly downregulated in SFCs, implying these biologic processes are inactive in these cells. All components of cell adhesion (PDLIM5, fc = 0.201; CALD1, fc = 0.281; DIAPH3, fc = 0.194; MACF1, fc = 0.741; PPME1, fc = 0.548; TMEM47, fc = 0.330; TLN1, fc = 0.410; PPME1, fc = 0.548) and cycle (CCND1, fc = 0.493; MAPK3, fc = 0.521; PIN1, fc = 0.656; CUL4B, fc = 0.425; CRIM1, fc = 0.043; THRA, fc = 0.676) were downregulated, most of which were connected with under-expressed MYC. Chromatin remodelers (CHD4, fc = 1.656; BAZ2B, fc = 1.800; and SMARCB1, fc = 1.272) associated with transcriptional hyperactivity were elevated in the network.

CSC marker and factors associated with stem cell maintenance, such as NOTCH1 (fc = 1.718), BCL9 (fc = 1.676), and SOX4 (fc = 1.683) were overexpressed. Stem cell intrinsic factors that control the reversible arrest of cell cycle were noted in this network. Over-expressed EGR1 and CHD4 and under-expressed MYC and CCND1 corresponded to the quiescent state of stem cells.

## Discussion

CSCs are thought to be the origin of recurrence and have the potential to survive chemotherapy. One feature of stem cells, cellular dormancy or quiescence, has increasingly gained interest as a relevant step in metastasis and chemoresistance.

Suspension culture systems used in this study stimulate symmetrical division and expand the stem cell compartment. In ovarian cancer, spheroids were first cultured from cancer cells obtained from peritoneal ascites in 2005 by Bapat et al. [13]. Subsequent studies reported that spheroids can be established from primary ovarian cancer tissues using this system [23]. The cancer cells which survived non-adherent substrates grew as spheroid cell clusters and presented features of CSC-like expression of CSC marker and tumorigenicity [24]. In our previous report, we also demonstrated that spheroids are enriched with cancer stem cells by showing the elevation of many stem cell markers, ALDH1, CD133, CD24, and SOX2 [12] that is in line with our current study.

In a suspension culture system for tumor cell spheroids, loss of adhesion and nutrient deprivation can promote growth arrest and cause CSCs to enter quiescence [2]. Our results are consistent with previous studies that suggested cell adhesion and cell cycle pathways were suppressed in quiescent or dormant tumor cells [4, 25].

Stem cells are transcriptionally active and express global regions of the whole genome rather than specific lineage genes [26]. This transcriptional hyperactivity is accompanied by chromatin-remodeling factors and general TFs [14]. Recently, quiescence has come to be regarded as actively regulated by TFs and epigenetic factors rather than dormant as a default [7]. In our study, both mRNA and miRNA analyses suggested that transcription-related functions were significantly hyperactivated relative to other specific functions (Tables 1, 2, 3, 4, 5, 6, 7 and 8). Chromatin remodelers (CHD4, BAZ2B, and SMARCB1) were also elevated in the network (Fig. 2). Considering the previous literature regarding spheroid culture in addition to the gene expression patterns of this study, our SFCs appeared to have more stem cell identity relative to parental cells from primary ovarian cancer.

In this study, EGR1 was the most over-expressed TF based on comprehensive analysis. Quiescent cells can exit or re-enter the cell cycle in response to environmental and cell intrinsic signs. EGR1 is an immediate response gene involved in cellular responses to stress and growth factors, and has been reported as an intrinsic regulator that promotes quiescence [6]. This can reduce tumor growth, but also leads to increased survival in response to stress signals in solid tumors. Mice with EGR1-KO presented an accelerated tumorigenesis in a two-step skin carcinogenesis study [15]. Meanwhile the phosphorylated form serves a protective function by inhibiting apoptosis of cells irradiated with UV [27].

The function of EGR1 is mediated with p53, a downstream tumor suppressor [15]. p53 is important in pathophysiology of ovarian cancer and high-grade serous ovarian cancers are ubiquitously TP53 mutant [28]. A p53 deficiency can promote cell cycle reentry through cell cycle-independent mechanisms [16, 29]. The role of EGR1 in chemoresistance was presented recently. In a study investigating properties of CSC, gene expression was compared between CSCs and cisplatin-resistant cells selected from lung cancer cells H460 [20]. There were close similarities between them, and EGR1 was one of the most significantly over-expressed genes in both cells. EGR1 enhances drug resistance by modulating MDR1 expression, according to a functional study [21].

The expression level of MYC, a master regulator, plays an important role in controlling the balance between proliferation and biosynthetic quiescence in stem cells [17]. There is a strict requirement for MYC activity to avoid cell death, and MYC inhibition induces stem cells to enter a quiescent state in various stem cell studies [18]. In hepatocellular carcinoma, MYC inactivation leads tumor cells to differentiate and many of them to die, but some cells showed stem cell properties and regained their proliferative capacity upon MYC reactivation [19]. In a mouse model, inactivation of MYC resulted in regression of liver tumors, and the tumor dormancy lasted for over 8 months.

MYC is necessary for quiescent cells to be activated rapidly from a poised state wherein multiple genes have initiated transcription but not elongated further. MYC controls transcriptional elongation mediated by RNA polymerase II, and its expression can lead to mRNA synthesis that promotes rapid proliferation [30]. The level of MYC is positively related to cell number, which was confirmed in spheroids from a glioma cell line [22]. Therefore, the reduced MYC expression in our ovarian SFCs may play a role in the maintenance of cellular quiescence suppressing proliferation.

Post-transcriptional regulation is emerging as a major contributor to quiescence biology. Under-expressed miR-130a-3p (previous ID: miR-130a) was a major alteration in our network, and had multiple connections to target genes, including over-expressed SOX4. In a previous study measuring mRNA stability of the quiescent transcriptome, targets of the miR-130 family were enriched in day seven contact-inhibited fibroblasts compared to proliferating cells [31]. The miR-130 family may have a potential role in promoting proliferation, or its target genes in maintaining a quiescent state in stem cells. miR-130a-3p is under-expressed in chemoresistant non-small cell lung cancer [32]. miR-130a-3p directly targets SOX4, which can upregulate ABCG2, a main contributing factor to multidrug resistance, and induce the reduction of cisplatin resistance.

In our network analysis, other regulators which are reported to be relevant in the molecular regulation of stem cells were also noted. PBX1, a stem cell reprogramming factor, has been observed to promote CSC-like phenotypes, including resistance to platinum in ovarian cancer cells [33]. Loss of PBX1 resulted in impaired self-renewal and quiescence in hematopoietic systems [34]. EP300 regulates transcription via chromatin remodeling as a histone acetyltransferase. Loss of EP300 was presented in multiple solid tumors, and was associated with increased tumorigenicity, CSC-like properties, and chemoresistance [35]. The roles of these TFs in our study were inconclusive because the directions of expression were opposite to those of previous studies.

Based on our results, the ovarian SFCs with quiescent features seem to be regulated by over-expressed EGR1, and under-expressed MYC and miR-130a-3p. These molecular mechanisms of ovarian CSC and its quiescence may provide insights into the biology of recurrence and metastasis. The novel agents affecting the key regulators may eradicate the chemoresistant quiescent lesion or activate minimally invasive disease to be susceptible for conventional chemotherapy. Our study could provide information for further studies to validate potential therapeutics.

There were limitations in our study. First, the number of cases was too small for our results to be applicable in general. Second, heterogeneity of cells could not be completely excluded. If ovarian CSCs could be sorted by FACS, surface markers might have been helpful in selecting CSCs with more precision. However, the usage is often limited due to low specificity of surface marker and low viability of sorted cells [36]. Third, because this study was designed to compare the gene expression of ovarian CSCs and parental cells, it is possible that the results were biased for stemness rather than common features of cancer such as proliferation and survival. Thus, the results should be interpreted with caution and validated with further functional and clinical studies. Forth, we performed and compared the gene expression profiles of cultured primary cells vs. spheroid-forming cells with same passage without data for initial epithelial tumor cells (passage 0). We could not completely exclude the possible alteration of gene expression of culture primary cells from the initial tumor cells.

## Conclusions

Two TFs, MYC and EGR1, and miR-130a-3p are hubs in the regulatory network of ovarian CSCs, where adhesion- and cell cycle-related genes were suppressed. The characteristic pattern of molecular expression in this study suggested that the SFCs remained in a quiescent state of CSC. Considering the clinical significance of a quiescent tumor populated with CSCs, our results may provide target molecules for further study to treat the chemoresistance and recurrence of ovarian cancer.

## Supplementary information

**Additional file 1 Table S1**. The Patient information. **Table S2**. The RNA QC result of 3 primary cancer cells and their corresponding spheroid forming cells. **Figure S1**. The Facs analysis for immune cells in representative primary tumor cells before and after Ber-Ep4 Dynabead treatment.

## Abbreviations

cDNA: Complementary DNA
CSC: Cancer stem cell
EGR1: Early growth response protein 1
EP300: E1A binding protein P300
miRNA: MicroRNA
mRNA: Messenger RNA
MYC: BHLH transcription factor
PBX1: Pre-B-cell leukemia homeobox 1
SFC: Spheroid-forming cell
SOX4: SRY (sex determining region Y)-related HMG-box 4
TF: Transcription factor
