# Supplementary material for: Integrative analysis of transcription factors and microRNAs in ovarian cancer cell spheroids
Source: J Ovarian Res. 2020 Feb 11;13:16. doi: 10.1186/s13048-020-00618-7 (PMC7014770; doi:10.1186/s13048-020-00618-7)
Supplement: Supplementary file 1 — Additional file 1 Table S1. The Patient information. Table S2. The RNA QC result of 3 primary cancer cells and their corresponding spheroid forming cells. Figure S1. The Facs analysis for immune cells in representative primary tumor cells before and after Ber-Ep4 Dynabead treatment. [file 13048_2020_618_MOESM1_ESM.docx]

Additional file 1

Table S1. The Patient information

| No | Dx * | Age | Site | Clilnical Stage | RNA QC ( Passage 2) | |
| --- | --- | --- | --- | --- | --- | --- |
|  |  |  |  |  | Parental | Spheroid |
| 1 | HGSC * | 58 | Ovary,primary | III | pass | pass |
| 2 | HGSC | 55 | Ovary,primary | IV | pass | pass |
| 3 | HGSC | 54 | Ovary,primary | III | pass | pass |
| 4 | HGSC | 72 | Ovary,primary | IV | pass | nonpass |
| 5 | LGSC | 46 | Ovary, primary | II | pass | pass |

* High grade Serous Carcinoma ; LGSC : Low grade serous carcinoma

Table S2. The RNA QC result of 3 primary cancer cells and their corresponding spheroid forming cells

| Sample | ug/uL | OD260/280 | OD260/230 | Total (ug) | Ratio(28S/18S) | RIN |
| --- | --- | --- | --- | --- | --- | --- |
| PC 1 | 0.4256 | 1.74 | 2.09 | 2.9793 | 1.9 | 9.2 |
| PC 2 | 0.3782 | 1.72 | 2.02 | 3.0255 | 2.0 | 9.9 |
| PC 3 | 0.4763 | 1.74 | 2.13 | 3.3338 | 2.1 | 8.5 |
| SFC 1 | 0.4248 | 1.70 | 2.01 | 3.3986 | 1.9 | 8.6 |
| SFC 2 | 0.3055 | 1.70 | 1.57 | 2.4442 | 1.9 | 9.5 |
| SFC 3 | 0.0797 | 1.74 | 1.52 | 0.2390 | 1.9 | 9.0 |

PC : Primary cancer cells, SFC : Spheroid forming cells

RIN (RNA Integrity Number): The quality of the total RNA by Agilent 2100 Bioanalyzer : The ratio of the magnification and peak pattern, 28s/18s ribosomal RNA. The recommended value for cDNA microarray analysis requires 7.0 or higher.

Figure S1. The Facs analysis for immune cells in representative primary tumor cells before and after Ber-Ep4 Dynabead treatment.

After single cell separation from ovarian tumor, the immune cells (CD45) comprises about 13% of the whole isolated cells (1x10^5), but after Ber-Ep4-coated dynabeads treatment, the proportion of CD45 positive cells becomes 0%.

**(CD45) isotype**

**Ovary primary cancer cells**


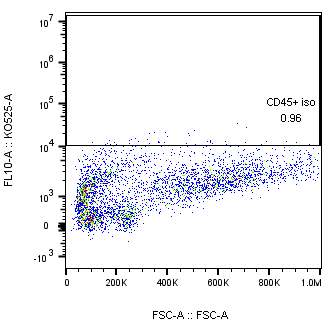

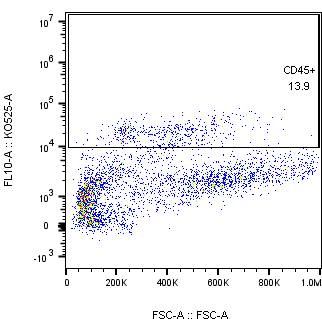

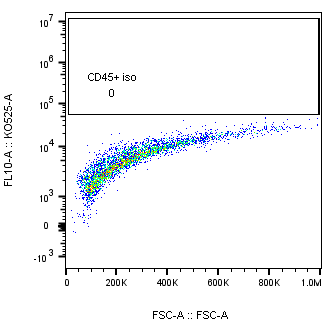

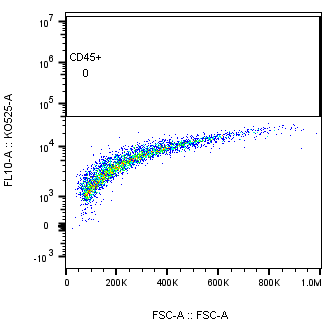


**Before**

**After**

**CD45**

**13.9%**

**0.96%**

**0.00%**

**0.00%**
